# Efficacy of probiotics in the prevention of diarrhea in ventilated critically ill ICU patients: meta-analysis of randomized control trials

**DOI:** 10.1186/s40560-021-00567-3

**Published:** 2021-10-15

**Authors:** Kentaro Shimizu, Tomoya Hirose, Hiroshi Ogura

**Affiliations:** Department of Traumatology and Acute Critical Medicine, Osaka University Graduate School of Medicine, 2-15 Yamadaoka, Suita, Osaka 565-0871 Japan

**Keywords:** Intensive care, Enteritis, Stool, Diarrhea, Probiotics, Mechanical ventilators, Critical care

## Abstract

We comment on the study by Batra et al. on the efficacy of probiotics in the prevention of ventilator-associated pneumonia in critically ill ICU patients. They also reported that probiotics administration was not associated with a statistically significant reduction in the incidence of diarrhea (OR 0.59; CI 0.34, 1.03; *P* = 0.06; *I*^2^ = 38%). However, their meta-analysis missed one RCT, and when we repeated the analysis including this RCT, we found that probiotics administration significantly reduced the incidence of diarrhea (OR 0.51; CI 0.28, 0.92; *P* = 0.02; *I*^2^ = 45.6%). We thus believe that probiotics administration is effective in reducing the incidence of diarrhea in ventilated critically ill ICU patients.

Dear Editor,

We read with interest the recently published article ‘Efficacy of probiotics in the prevention of VAP in critically ill intensive care unit (ICU) patients: an updated systematic review and meta-analysis of randomized control trials’ by Batra et al. [1] They showed that the administration of probiotics reduced the incidence of ventilator-associated pneumonia (VAP), the duration of mechanical ventilation, length of ICU stay, and in-hospital mortality, but that it was not associated with a statistically significant reduction in the incidence of diarrhea (odds ratio [OR] 0.59; confidence interval [CI] 0.34, 1.03; *P* = 0.06; *I*^2^ = 38%).

Probiotics are live non-pathogenic microbes that reduce bacterial translocation by activating mucosal immunity. Probiotics increase short-chain fatty acids and suppress systemic inflammatory response by stabilizing the gut microbiota [2]. The immune system’s T cells and B cells are influenced by the microbiota and could be related to immune-related and miscellaneous diseases [3]. This mechanism might indicate the importance of maintaining the gut microbiota by probiotics to help suppress VAP. Therefore, it is presumed that the incidence of diarrhea would also decrease. In our past propensity study, probiotics reduced the incidence of both diarrhea and VAP [4].

We scrutinized the nine randomized controlled trial papers examined in the Batra et al. review article and identified an issue. The incidence of diarrhea of Shimizu et al. [5] was not included in the meta-analysis by Batra et al. [1]. In that paper, enteritis was defined as the acute onset of continuous liquid stools for more than 12 h. The incidence of enteritis was significantly lower in the probiotics administration group than that in the control group (6.3% vs. 27.0%; *P* < 0.05).

Therefore, we reassessed the meta-analysis by adding the Shimizu et al. paper [5] to the four papers included in the Batra et al. paper that examined diarrhea. As a result, five studies reported diarrhea in 526 patients (264 in the probiotic arm and 262 in the placebo arm). We found that the administration of probiotics significantly reduced the incidence of diarrhea (OR 0.51; CI 0.28, 0.92; *P* = 0.02; *I*^2^ = 45.6%), as shown in the Fig. 1.

**Fig. 1:**
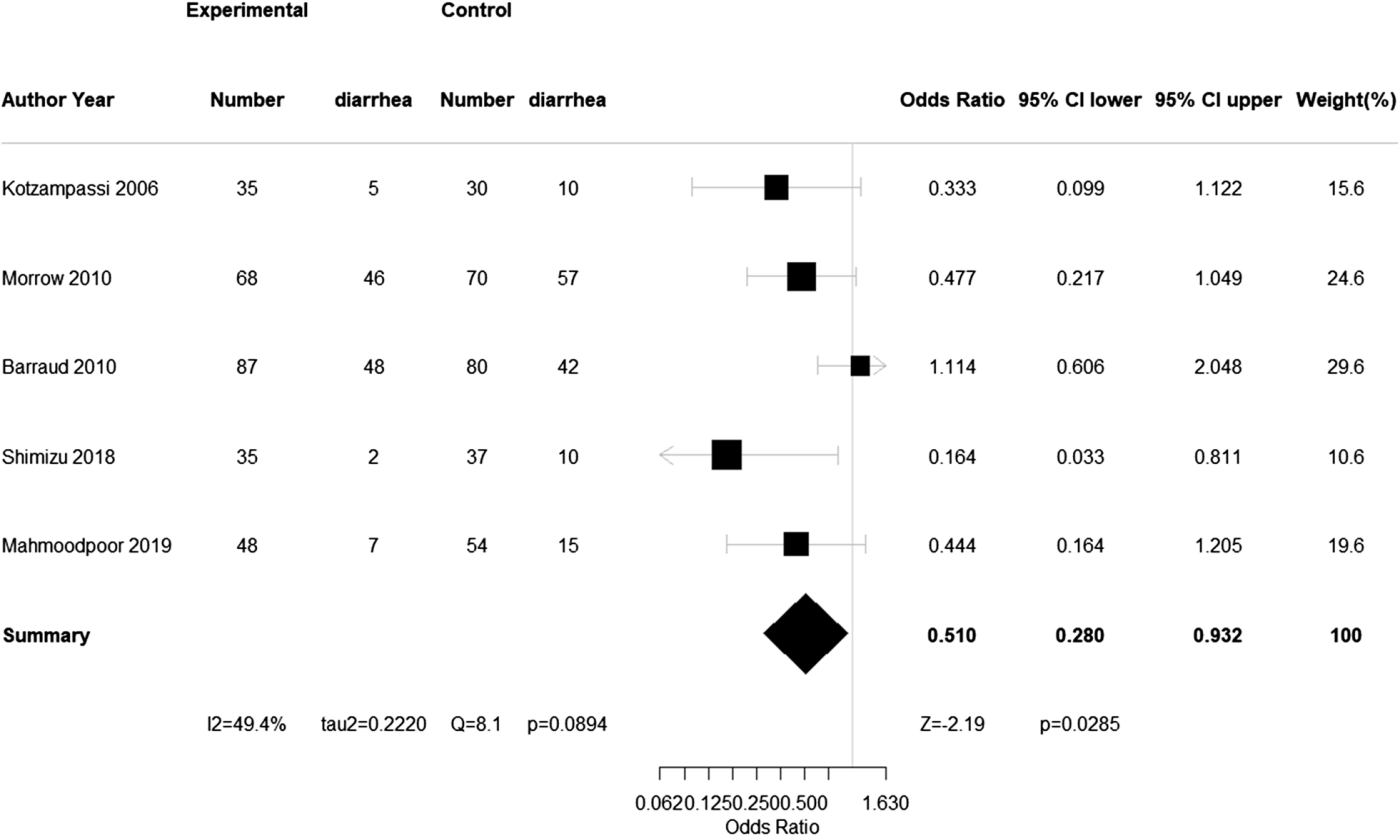
Forest plot of the studies that examined the incidence of diarrhea

This result indicated an effect of probiotics on reducing diarrhea in ventilator-treated patients. Probiotics have been reported to reduce infectious complications related to postoperative complications [6] and trauma [7] and to reduce *Clostridium difficile* infection induced by antibiotics [8]. The mechanism is thought to be maintenance of immunity by stabilizing the gut microbiota. Patients who require mechanical ventilation are also at high risk of deterioration of their intestinal bacterial flora due to critical illness such as sepsis and the administration of antibiotics [9], which could induce diarrhea and VAP. In fact, the administration of probiotics for such conditions induced significant production of acetic acid, a metabolite of total obligate anaerobes and gut microbiota [2]. It is reasonable that the maintenance of the gut microbiota not only reduces VAP, but also contributes to a reduction of diarrhea. We thus believe that the administration of probiotics is effective in reducing the incidence of diarrhea in ventilated critically ill ICU patients.

## Data Availability

Not applicable.

## Abbreviations

CI: Confidence interval
ICU: Intensive care unit
OR: Odds ratio
VAP: Ventilator-associated pneumonia
